# Managing Vulvodynia with Central Sensitization: Challenges and Strategies

**DOI:** 10.3390/jcm12113851

**Published:** 2023-06-05

**Authors:** Cristina Rubal, Augusto Pereira, Laura Calles Sastre, Belén Almoguera Pérez-Cejuela, Sofía Herrero Gámiz, Pilar Chaves, Tirso Pérez Medina

**Affiliations:** 1 Department of Obstetrics and Gynecology, Autonoma University of Madrid, 28222 Madrid, Spain; rubalcristina@gmail.com; 2 Department of Gynecologic Surgery, Puerta de Hierro University Hospital, 28222 Madrid, Spain; lauracall@hotmail.com (L.C.S.); almoguerabelen@gmail.com (B.A.P.-C.); sofiaherrerogamiz@gmail.com (S.H.G.); pilar.chape@gmail.com (P.C.); tirsoperezmedina@gmail.com (T.P.M.)

**Keywords:** vulvodynia, vulvar pain, vestibular pain, perineal pain, chronic pelvic pain, central sensitization, treatment

## Abstract

**Background**: Vulvodynia is defined as a chronic idiopathic vulvar pain condition. This study aimed to investigate the effect of central sensitization on the prognosis of neuromodulator treatment for vulvodynia. **Method:** A total of 105 patients with vulvodynia who underwent pelvic mapping pain exploration were included and scored according to the Convergence PP Criteria for pelvic pain and central sensitization. The patients were treated according to chronic pelvic pain guidelines, and their response to treatment was evaluated. **Results:** A total of 35 out 105 patients (33%) with vulvodynia had central sensitization, which was associated with comorbidities, dyspareunia, pain with micturition, and pain with defecation. Dyspareunia and pain with defecation were independent prognostic factors for central sensitization. Patients with central sensitization experienced more pain during intercourse, urination, or defecation, had more comorbidities, and responded worse to treatment. They required more treatment, with a longer response time (over 2 months). Patients with localized vulvodynia were treated with physiotherapy and lidocaine, while patients with generalized vulvodynia were treated with neuromodulators. Amitriptyline was effective in treating patients with generalized spontaneous vulvodynia and dyspareunia. **Conclusions:** Overall, this study highlights the importance of considering central sensitization in the diagnosis and treatment of vulvodynia and the need for individualized treatment based on the patient’s symptoms and underlying mechanisms. Vulvodynia patients with central sensitization had more pain during intercourse, urination, or defecation, and responded worse to treatment, requiring more time and medication.

## 1. Introduction

Vulvodynia is a chronic idiopathic vulvar pain lasting at least 3 months, firstly introduced as a separate entity from vulvar pain syndrome in 2015 by the International Society for the Study of Vulvovaginal Disease (ISSVD) [1].

It is the most common cause of pain during sexual intercourse in postmenopausal women [2]. The prevalence is between 3 and 15% [3], although it is difficult to assess due to its heterogeneous presentation form, frequent association with other comorbidities, and neuromodulation through anxiety status or estrogen deficiency [4]. The most frequent clinical presentation is pain on contact with the vestibule due to sexual intercourse (dyspareunia) and pain with the use of tampons or tight clothing (allodynia) [3,5,6].

The coexistence of symptoms of neuropathic pain (perineal allodynia, burning, paresthesia), pain with or after micturition and/or defecation, variability in the distribution or intensity of pain, comorbidities, and trigger point pain in the pelvic floor musculature may be suggestive of central sensitization [7]. Central sensitization is an increase in the excitability of the central nervous system in which normal inputs evoke exaggerated responses [8]. It is a process resulting from increased neuronal membrane excitability and synaptic efficacy due to the nervous response to inflammation and neuronal damage. It is a phenomenon of synaptic neuroplasticity based on altered sensory processing in the brain, dysfunction in descending pain inhibitory mechanisms, increased activity in pain pathways, and long-term potentiation of synapses in the anterior cingulate cortex [9,10]. When the noxious stimulus induces tissue damage in the pelvic organs, it activates specialized nociceptors which are transduced into impulses transmitted via A delta (fast, myelinated) and C fibers (slow, unmyelinated) via the hypogastrius plexus and pudendal nerves. The descending pathways from the rostral ventromedial medulla and the periaqueductal gray can modulate the pain afferent pathways, augmenting or surprising the sensory input, playing an important role in hyperalgesia. The rostral ventromedial medulla could also facilitate nociceptive processing and transmission maintaining hyperalgesia after the peripheral tissue damage. There is also an important influence of visceral cross-sensitization involving the dorsal root ganglion. Therefore, there are central and peripheral mechanisms for the maintenance of pain. Peripheral sensitization happens when sensory impulses travel antidromically through the afferent fibers and produce neurogenic inflammation, involving the degranulation of mast cells and neuropeptides substance *p* and calcitonin related peptide, in the area innervated by the primary afferent nociceptors. This has been demonstrated to happen in some of the comorbid diseases to vulvodynia (irritable bowel syndrome, interstitial cystitis…). Peripheral sensitization can be the beginning of central sensitization, when pain persists after the resolution of the cause. This may be because of substance *p* and calcitonin gene-related peptide (CGRP) and the increased reactivity of N-methyl-D-aspartate (NMDA) and amino-3-hydroxy-5-methyl-4-isoxazole propionate (AMPA) receptors increasing the signals received by the central nervous system, leading to hyperalgesia and allodynia. Thus, pain is uncoupled from the noxious stimulus, and normal inputs evoke exaggerated responses [10,11].

Vulvodynia is understudied, due both to the complexity of generalized pain disorders as a whole and the hesitance of patients to share their symptoms to clinicians because of the stigma behind female pain [12]. Furthermore, the precise etiology of vulvodynia is still unknown, but several theories claim that it has a multifactorial origin (embryonic, chronic inflammatory syndromes, environmental factors, contact with irritants, recurrent vulvovaginal infections) [13]. It has been demonstrated that the painful area presents a proliferation of nerve fibers and chronic inflammation [14]. However, the role of central sensitization in chronic pelvic pain, and how patients affected with this pathology would respond to the typical treatment of this pain compared to those who are not affected by it, is still uncertain. There is also insufficient evidence on the efficacy of the most frequently used therapeutic steps, as most of these uses are based on trial and error [12].

The aim of this study was to evaluate the response to commonly used treatments in vulvodynia, including physiotherapy, topical lidocaine, intravaginal diazepam, antidepressants, anticonvulsants, and minimally invasive neuromodulation, and compare the outcomes between patients with and without central sensitization. The study also aimed to examine the demographic and treatment disparities among patients with generalized and localized vulvodynia, as well as those with and without central sensitization. Finally, the study further sought to ascertain whether central sensitization exacerbates or delays treatment response and its prognostic value for vulvodynia, as well as to explore whether the type and characteristics of vulvodynia impact the prescribed and successful treatment approaches.

## 2. Materials and Methods

### 2.1. Patients

The medical records of 393 consecutive female patients with chronic pelvic and perineal pain syndrome who sought consultation at the Puerta de Hierro Majadahonda University Hospital in Madrid, Spain, from May 2018 to July 2022 were reviewed. Ethical approval for this study was granted by the Puerta de Hierro Majadahonda University Hospital Institutional Review Board. 

The main inclusion criteria were female patients with chronic pelvic and perineal pain syndrome that had been experiencing vulvar, vestibular, vaginal, clitoral, and/or perineal pain for at least three months diagnosed with vulvodynia [3]. According to the IVSSD, vulvodynia was defined as vulvar pain of at least 3 months’ duration, with no clear identifiable cause, with potential associated factors, and provoked by trigger point contact. Patients with vulvodynia were classified as:

a. Localized: vestibulodynia (pain in vaginal vestibule), clitoridynia (pain in clitoris); generalized; or mixed (localized and generalized).
b. Provoked: insertional, contact; spontaneous (no contact); or mixed (provoked and spontaneous).
c. Onset: primary (present from the first sexual contact or insertion of a tampon) or secondary (present after a period of asymptomatic sexual contact).
d. Temporal pattern: intermittent, persistent (if symptoms have lasted more than 3 months and persist), constant, immediate (during physical contact), delayed (symptoms appear later).

The exclusion criteria referred to patients with pain in a location other than those indicated above, with a symptom onset below 3 months, a diagnosis of any type of known-cause vulvar pain (the excluded causes being obstetric, traumatic, surgical, or dermatological), patients with no programmed follow-up consultations, patients with no prescribed treatment, or patients with incomplete data. 

### 2.2. Data Assessed

The data assessed included age on first consultation, complete medical history (gynecological, obstetric, and surgical history), pain location (vulvar, vaginal, perineal, urethral meatus, vestibular, multifocal (more than 2 locations), old obstetric scar), duration of pain (estimated by the patient as the number of years between the onset of symptoms and the date of first consultation), comorbidities, dyspareunia, type of vulvodynia (localized/generalized and provoked/spontaneous vulvodynia), result on the Convergences PP Criteria Score, treatment before consultation, treatment prescribed after consultation, number of physiotherapy sessions, and subjective response to prescribed treatment (evaluated by a percentage of relief of their symptoms).

The comorbidities were selected according to Yunus et al. [15], related to the concomitant presence of several clinical hypersensitivity syndromes, such as fibromyalgia, interstitial cystitis, irritable bowel syndrome, migraine, endometriosis, anxiety and depression, neuropathies, temporomandibular joint dysfunction, chronic fatigue syndrome, and/or a history of multiple chemical sensitivity.

The Convergence PP Criteria Score was developed by Levesque et al. [7] as clinical criteria of central sensitization for patients with chronic pelvic and perineal pain. They were calculated for each patient, evaluating the presence of: (1) pain influenced by distention and/or bladder emptying, (2) pain influenced by distention and/or rectal emptying, (3) pain during sexual intercourse, (4) perineal or vulvar allodynia, (5) pelvic trigger points (piriformis muscle, obturator internus muscle, and/or levator ani muscles), (6) pain after urination, (7) pain after defecation, (8) pain after sexual intercourse, (9) variability in pain distribution or intensity, (10) comorbidities (fibromyalgia, chronic fatigue syndrome, post-traumatic stress disorder, chronic temporomandibular joint pain, sensitivity to chemicals, restless legs syndrome). The score ≥ 5/10 was considered suggestive of central sensitization. 

### 2.3. Exploratory Procedures or Pain-Mapping Method

The examination was performed by a single physician with expertise in chronic pelvic pain and data from the examination were collected based on the pain-mapping method reported by Pereira et al. [16], including the following.

#### 2.3.1. An S2–S4 Neurological Examination

- Cotton swab testing of the S2–S4 dermatome and vestibule: the absence of signs and symptoms during the physical examination confirms the integrity of the C fibers.
- Clitoris, bulbospongiosus, and perineal reflexes: an evaluation of the motor response of the terminal branches of the pudendal nerve is conducted by gently touching the labium minus lateral to the clitoris, the perineum, and the clitoris with a cotton swab, and the normal motor activity at S2–S4 is indicated by anal sphincter contraction.
- Tinel’s sign in the sciatic spine area: to evaluate the third segment of the pudendal nerve, pain is reproduced with transrectal compression of the third segment of the PN (Tinel sign) in the sciatic spine and Alcock’s canal.
- Tinel’s sign at the clitoris: to evaluate the dorsal nerve of the clitoris, the clitoris is compressed to locate painful spots.

#### 2.3.2. Exploration of the Pelvic Girdle

Bilateral palpation in order to identify painful spots—retropubic, ischiopubic ramus, ischium, sacrospinous ligament, sacrum, and coccyx area. Bilateral mobility of the hip and lower extremities is explored (abduction, extension, flexion, and external and internal rotation).

#### 2.3.3. Exploration of Pelvic Floor Muscles

- Levator ani muscle (LAM): assessment of painful palpation of the pubococcygeus muscle.
- Obturator internus muscle (OIM): contracture of the OIM with flexion and external rotation of the hip in the supine decubitus position and transgluteal examination of OIM segments—pelvic (ischium), medium (midpoint between trochanter and coccyx), and gluteal (hip).
- Piriformis muscle (PM): simultaneous hip external rotation and abdominal flexion is encouraged to reproduce the pain. PM is palpated transgluteally five centimeters above the OIM middle segment.

Patients were asked to rate their level of pain on a scale from 0 (no pain) to 10 (worst pain ever experienced) during the assessment.

### 2.4. Protocols for Vulvodynia Treatment

The patients in this study were treated according to the recommendations on the National Guideline Clearinghouse for pharmacological management of neuropathic pain and recent vulvodynia studies. The first step of treatment involved dietary recommendations, topical medications (such as lidocaine, which blocks transmission of afferent C fibers and treats peripheral sensitization), physiotherapy, and psychological therapy [12]. If these treatments were not effective, the second step involved the use of tricyclic antidepressants, such as amitriptyline, which have been shown to decrease pain in patients with a neuropathic component. The third step involved minimally invasive neuromodulation, with or without botulinum toxin, and was generally prescribed to patients with generalized spontaneous vulvodynia (GSV) and central sensitization [12,17,18,19,20].

- Patients with provoked vulvodynia could be prescribed:
  ▪ Pelvic floor physiotherapy, including 30–40 min of intravaginal and external perineal massage (along the OIM, EAM, and PM), thermotherapy, and biofeedback techniques such as the use of vibrators and vaginal dilators to use during the session and at home. If lack of response or partial response to the described techniques was experienced, transcutaneous electrical nerve stimulation (TENS) was used 2 times a week for 20 min each session, and the electrodes were applied to the labia majora in a V-shape.
  ▪ Topical lidocaine (5% lidocaine clorhidrate gel, 3–4 applications/24 h).
  ▪ Vaginal diazepam (5 mg diazepam ovules, 1 ovule/48 h).
- Patients with spontaneous vulvodynia could be prescribed:
  ▪ Neuromodulators such as tricyclic depressants (amitriptyline, 25 mg/24 h) and serotonin and noradrenaline reuptake inhibitors (duloxetine, 30–60 mg/24 h).
  ▪ Anticonvulsants (gabapentin, 300 mg/8 h and pregabalin, 75 mg/12 h).
  ▪ Minimally invasive neuromodulation (MIN) techniques (infiltration of impar ganglion, pudendal nerve, and 100 units of onabotulinum toxin A, pulsed radiofrequency (PRF) of sacral roots and pudendal nerve).
- All types of vulvodynia could be prescribed:
  ▪ Psychological therapy (normally cognitive behavioral) for treatment of stress and the response to pain when a psychological component to pain was identified.
  ▪ Hygienic–dietary and behavioral measures, such as vulvar hygiene, lubrication, and use of vaginal dilators.
- Patients with vaginal atrophy could be prescribed:
  ▪ Topical estradiol creams for menopausal women.
  ▪ Ospemifene (60 mg/24 h).

In the follow-up consultation, the response to treatment was evaluated using the patient’s subjective relief of symptoms as a percentage, using a subjective pain evaluation scales (EVA). A relief of symptoms greater than 30% was considered a response to the prescribed treatment. This was done in order to exclude the possibility of a placebo effect. 

### 2.5. Statistical Analysis

Descriptive analysis was carried out to evaluate the cohort. Dichotomous or categorical variables were expressed as absolute values and percentages; continuous variables were expressed as the mean and median. The Chi-square test was used to estimate the relationship between variables and pain. The relative risk (RR) was calculated. Differences in distributions of dichotomous variables were analyzed using Fisher’s exact test. Differences in distributions of continuous variables were analyzed using the Kruskal–Wallis test. A value of *p* ≤ 0.05 was considered statistically significant. 

Kaplan–Meier curves were used to compare treatment efficacy and treatment time, to determine the number needed to treat, number of patients who must be treated to obtain cure of their disease, and to evaluate the response to neuromodulator treatment between patients with central sensitization and patients without central sensitization [16]. Subsequent to the univariate study, all variables with *p* < 0.1 entered a multivariate study by performing a Cox logistic regression to determine if central sensitization was an independent prognostic factor [21]. The statistical analysis was performed using IBM SPSS Statistics (IBM Corp. Released 2022. IBM SPSS Statistics for Windows, Version 29.0. Armonk, NY, USA: IBM Corp.).

## 3. Results

### 3.1. Patients

We identified 393 medical records in our hospital’s electronic database of female patients who sought consultation between May 2018 and July 2022. After excluding 288 cases that had missing data needed for the study or met exclusion criteria, a total of 105 patients met all inclusion criteria and were selected for evaluation.

### 3.2. Data Assessed

Demographic characteristics of patients are shown in Table 1 and Table 2. Regarding the obstetric data, 52 patients (49.5%) were nulligravid, 18 patients (17.1%) were primiparous, 32 patients (30.5%) were multiparous, and 3 (2.9%) had no available obstetric data. Of them, 10 patients (9.5%) had received a cesarean (primiparous: 3, and multiparous: 7). The mean age of the cohort was 40.9 (range 15–74). The mean pain duration was 3.7 years (range: 0.1–30), and the mean PP criteria score was 4.1 (range: 1–7). The most common pain location was multifocal (41%), followed by vaginal (17.1%), vulvar (15.2%), and vestibular (12.4%). The main diagnosis was generalized and spontaneous vulvodynia (45.3%), followed by localized and provoked vulvodynia (20.8%). A total of 53.3% of patients had been previously treated, and 92.4% were prescribed a treatment. The most frequently prescribed treatments were physiotherapy (71.4%), intravaginal diazepam (49.5%), minimally invasive neuromodulation (46.7%), psychological therapy (31.7%), and topical lidocaine (30.5%); results are shown in Table 3 and Table 4.

Of the 105 patients analyzed, 35 (33.3%) fulfilled five or more of the Convergence PP Criteria, this being the point suggested by Levesque et al. to consider central sensitization. A total of 40% of patients had any of the comorbidities described above, 91.4% had dyspareunia, 37.1% pain with defecation, and 77.1% pain with micturition. The mean score obtained on the PP score among patients with no central sensitization was 4.1, which remained low for patients who developed central sensitization (5.6 points). Results are shown on Table 1.

The muscular distribution of pain in vulvodynia is in the LEA, OIM, and PM muscles [15]. In our study, 100 patients (92.4%) displayed pain in at least one of those pelvic trigger points. Out of the patients with central sensitization, 100% displayed pain in those pelvic muscular trigger points, as well as 92.6% of patients without central sensitization. The hip examination and lower extremity mobility were found to be normal.

A number of 80 patients attended a follow-up consultation, from which a subjective relief to pain was recorded. The mean follow-up for these patients was 248.6 days (range 0–1449). Our results have revealed improvements in 63.8% of patients who attended a follow-up consultation. The response to the prescribed treatments according to the type of vulvodynia was as follows: generalized and spontaneous vulvodynia displayed 66.5% response; localized and provoked vulvodynia, 78.9%; localized and spontaneous vulvodynia, 73.6%; and generalized and provoked vulvodynia, 63.1%. All results according to the administered treatment are detailed in Table 5.

### 3.3. Statistical Analysis

This study highlighted demographic differences among different patient groups. Univariate analysis also found that central sensitization (PP score > 5), was correlated with various factors, including comorbidities (*p* = 0.006), dyspareunia (*p* = 0.004), pain with defecation (*p* < 0.001), pain with micturition (*p* < 0.001), and treatment (*p* = 0.018). However, logistic regression analysis revealed that pain with defecation (*p* = 0.010) and dyspareunia (*p* = 0.028) were the only independent prognostic factors for the development of central sensitization; results are shown in Table 1 and Table 3. 

Among different types of vulvodynia, the univariate analysis found significant differences in relation to age (*p* < 0.001), pain location (*p* < 0.001), dyspareunia (*p* = 0.028), pain with defecation (*p* = 0.012), and pain in pelvic trigger points (*p* = 0.065). However, only the latter was found statistically significant (*p* = 0.006) in the multivariate analysis, as shown in Table 2. 

Thus, pain with defecation and pain with micturition had an independent influence on the prognosis of the development of central sensitization and there was a significant difference between vulvodynia types in patients with pain in pelvic trigger points.

Additionally, the study also found significant associations between quantitative variables, such as age and type of vulvodynia (*p* < 0.001), and in the PP criteria score between patients with a duration of symptoms larger and lower than 2 years (*p* = 0.021); results are shown in Table 2. This study demonstrated a weak direct correlation between the score and the duration of symptoms (*p* = 0.024). 

Furthermore, this study analyzed treatment responses and found that patients without central sensitization responded more frequently to treatment for provoked vulvodynia (76.5%) than for spontaneous vulvodynia (64.7%) (*p* = 0.04). However, there was no significant difference in response to treatment between those groups in patients with central sensitization (*p* = 0.631). Patients with central sensitization were prescribed more lidocaine, minimally invasive neuromodulator treatments, and psychological therapy, as shown in Table 4. The number needed to treat (NNT) when having comorbidities or dyspareunia was 3.3 patients, pain with micturition 1.6, and pain with defecation 2.4. 

**Figure 1:**
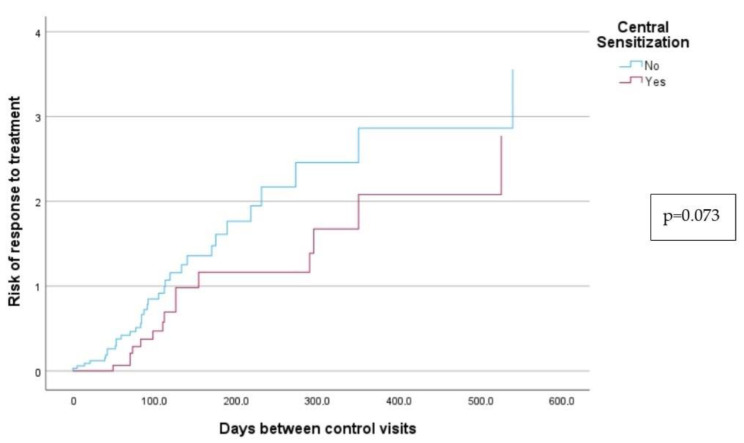
Kaplan–Meir Curves to analyze the response to treatment in patients with and without central sensitization.

Finally, Kaplan–Meier curves showed that patients with central sensitization took longer to respond to treatment than those without central sensitization (64, 6 days, respectively), but the difference was not statistically significant (*p* = 0.073). The results of this analysis are shown in Table 5 and Figure 1. This study also demonstrated a weak inverse correlation between the PP criteria score and the response to treatment (*p* = 0.009).

## 4. Discussion

Vulvodynia is poorly understood and poorly reported [22]. Its exact etiology is yet to be pinpointed; some authors suggest that genital infections, contact dermatitis, or irritants could be the culprits, but findings have been contradictory [23,24]. In patients with vulvodynia, with or without central sensitization, there is a commonality in the peripheral sensitization of C fibers in the vestibule, trigger points in the pelvic musculature, and neuropathic pain. The key differences lie in the presence of viscerosensitization, leading to pain with sexual intercourse, micturition, or defecation, as well as the presence of comorbidities.

Our study revealed several demographic and treatment differences among patients with generalized and localized vulvodynia, as well as between vulvodynia with and without central sensitization. Our study showed that approximately one-third of participants developed central sensitization, and based on our findings, we can establish a phenotype of patients with vulvodynia and central sensitization, which is correlated with age, symptom duration, pain location, and comorbidities.

Patients with central sensitization tended to be older, with a mean age of 43.5 years at their first consultation. Significant differences were observed between age groups and pain location (*p* = 0.033), and between age and type of vulvodynia (*p* < 0.001), with multifocal pain being the most common presentation (*p* < 0.001). Specifically, younger patients were more likely to have localized provoked vulvodynia, while patients with spontaneous generalized vulvodynia tended to be older, with a difference of 17 years.

Patients with central sensitization tended to have a longer duration of symptoms (almost 5 years). In our study, there was also a difference in the PP criteria score between patients with lower (score of 3.8) and higher (score of 5) symptom duration (*p* = 0.021), with a weak direct correlation between the score and symptom duration demonstrated (*p* = 0.024). Furthermore, significant variations in PP score were observed when symptom duration exceeded 2 years (*p* = 0.021).

Logistic regression analysis revealed that patients with central sensitization were five times more likely to experience dyspareunia and three times more likely to experience pain on defecation than other patients. This finding suggests that the presence of dyspareunia or proctalgia is an independent prognostic factor in the development of central sensitization in patients with vulvodynia.

Finally, patients with GSV had a higher number of comorbidities compared to other types of vulvodynia, suggesting that this form of vulvodynia may have a more complex and multifactorial pathophysiology.

Our study included a total of 105 patients, of whom 97 were prescribed a treatment plan. Among these patients, 80 attended follow-up consultations for a period of over 8 months, and 63.8% (51 out of 80) reported a significant improvement in their symptoms. However, the response to treatment was influenced by two significant factors described above: the presence of central sensitization of pain and the type of vulvodynia.

Patients with central sensitization had a lower response rate to treatment compared to others and required three times more treatment than patients without central sensitization (*p* = 0.053). Additionally, there was an inverse correlation between the PP score and response to treatment (*p* = 0.009), indicating that patients with higher scores had a lower response to treatment. Conversely, the PP score showed a direct correlation with the duration of symptoms.

With regard to the type of vulvodynia, patients with generalized vulvodynia had a lower response rate to treatment (65.7%) compared to patients with localized vulvodynia (76.6%) (*p* = 0.052). Patients without central sensitization responded more frequently to treatment for provoked vulvodynia (76.5%) than for spontaneous vulvodynia (64.7%). However, there was no significant difference in response to treatment between patients with provoked or spontaneous vulvodynia who had central sensitization (*p* = 0.631).

Around 60% of patients with central sensitization were treated with amitriptyline, particularly those with GSV and dyspareunia. Other frequently prescribed agents were topical lidocaine (*p* = 0.05), minimally invasive neuromodulation (*p* = 0.007), and psychological therapy (*p* = 0.015).

Finally, the response time to treatment was longer for patients with central sensitization (293 days) compared to patients with vulvodynia but no central sensitization; the time period for response to treatment was set at more than 2 months, and the number of patients needed to treat when having comorbidities or dyspareunia was 3.3, with pain related to micturition at 1.6, and with defecation at 2.4.

Despite the many unknown aspects of vulvodynia, its importance should not be underestimated. Recent studies have shown that it significantly affects quality of life, including the ability to perform basic activities and experience sexual satisfaction [25]. Some studies have indicated that treatment, especially physical therapy, can improve symptoms, while others have shown that a placebo can be as effective as conventional treatments [26]. The mean score of PP criteria for patients with central sensitization and vulvodynia is lower and the frequency is lower than other patients with chronic pelvic pain and central sensitization [16], which should be investigated. 

While this study provides valuable insights into the demographic, clinical characteristics, and treatment of patients with vulvodynia, its small sample size and lack of diversity may limit the generalizability of the findings. As it is a study based on cohorts, there are limitations when studying rare illnesses, which is the case for vulvodynia, which is rarely reported by patients [22]. Moreover, there is a high possibility of loss of patient data as fewer and fewer patients attend the follow-up appointments. Additionally, the use of self-reported measures and the absence of a control group could introduce bias into the results. Despite these limitations, this study highlights the importance of identifying central sensitization as a key factor in the development and treatment of vulvodynia, and suggests that amitriptyline may be a useful treatment option for patients with generalized spontaneous vulvodynia and dyspareunia. However, future efforts should focus on increasing awareness of vulvodynia among both the general public and healthcare professionals, diversifying samples, and improving the designs of clinical trials to further investigate treatment options and potential risk factors.

## 5. Conclusions

Prescribed treatments for patients with vulvodynia (with and without central sensitization) were multidisciplinary in nature, combining several treatments simultaneously. The prescribed treatments had a response rate of 60.9% for patients with central sensitization, which was lower compared to patients without central sensitization (72.7%). The most frequently used medications for patients with central sensitization were diazepam (37.5%), amitriptyline (40.3%), lidocaine (42.5%), anticonvulsants (42.9%), MIN (51.5%), along with physiotherapy (33.3%) and psychological therapy (58.8%). Furthermore, central sensitization delayed the treatment response, and patients required more treatment, with a longer response time (over 2 months). Patients with vulvodynia and central sensitization experienced more pain during intercourse, urination, and defecation, and they had a higher prevalence of comorbidities. Dyspareunia and proctalgia were independent prognostic factors for central sensitization. In terms of the type of vulvodynia, patients with generalized vulvodynia had a lower treatment response rate compared to patients with localized vulvodynia, and those without central sensitization responded more frequently to treatment for provoked vulvodynia than spontaneous vulvodynia. However, the small sample size, the lack of a control group, and the lack of diversity in the study may limit the generalizability of the findings.

## Figures and Tables

**Table 1:** Correlation between variables and patients with and without central sensitization.

Demographics	Overall N = 105	CS N = 35	No CS N = 70	UA *p*	HR	CI 95%	MA *p*
Age, years, mean (range)	40.9 (15–74)	43.5	39.6	0.195	-	-	-
Duration of pain, years, mean (range)	3.7 (0.1–30)	4.9	3.1	0.088	1.011	0.946–1.080	0.748
PP score							
Mean (range)	4.1 (1–7)	5.6	3.3	-	-	-	-
Pain location, N							
Multifocal	43 (41.0%)	13 (37.1%)	30 (42.9%)	0.132	-	-	-
Vaginal pain	18 (17.1%)	6 (17.1%)	12 (17.1%)				
Vulvar pain	16 (15.2%)	3 (8.6%)	13 (18.6%)				
Vestibular pain	13 (12.4%)	6 (17.1%)	7 (10.0%)				
Clitoral pain	7 (6.7%)	1 (2.9%)	6 (8.6%)				
Perineal pain	5 (4.8%)	4 (11.4%)	1 (1.4%)				
Urethral meatus	2 (1.9%)	1 (2.9%)	1 (1.4%)				
Old obstetric scar	1 (1.0%)	1 (2.9%)	0 (0%)				
Vulvodynia Type, N							
GSV	48 (45.3%)	19 (54.3%)	29 (41.4%)	0.525	-	-	-
LSV	17 (16.0%)	6 (17.1%)	11 (15.7%)				
GPV	18 (17.0%)	5 (14.3%)	13 (18.6%)				
LPV	22 (20.8%)	5 (14.3%)	17 (24.3%)				
Comorbidities	25 (23.8%)	14 (40.0%)	11 (15.7%)	**0.006**	0.360	0.049–2.634	0.193
Dyspareunia	78 (74.3%)	32 (91.4%)	46 (65.7%)	**0.004**	5.088	1.188–21.791	**0.028**
Pain with micturition	36 (34.3%)	27 (77.1%)	9 (12.9%)	**<0.001**	1.840	0.813–4.164	0.139
Pain with defecation	19 (18.1%)	13 (37.1%)	6 (8.6%)	**<0.001**	3.131	1.309–7.490	**0.010**
Pain in pelvic trigger points	100 (95.2%)	35 (100%)	65 (92.6%)	0.125	-	-	-

Abbreviations: CS: central sensitization; N: number of patients (%); HR: hazard ratio; CI 95%:confidence interval 95%; UA: univariate analysis; MA: multivariate analysis; *p*: statistical significance PP score: Convergences PP Score; GSV: generalized and spontaneous vulvodynia; LSV: localized and spontaneous vulvodynia; GPV: generalized and provoked vulvodynia; LPV: localized and spontaneous vulvodynia. Bold: significant number.

**Table 2:** Correlation between variables and vulvodynia types.

Demographics	GSV N = 48	LSV N = 17	GPV N = 18	LPV N = 22	UA *p*	HR	CI 95%	MA *p*
Age, years, mean	47.4	40.0	38.8	29.1	**<0.001**	0.989	0.961–1.019	0.478
Duration of pain, years, mean	3.8	2.6	4	4.2	0.807	-	-	-
PP score								
Mean (range)	4.4	4.0	3.7	3.8	0.859	-	-	-
Pain location, N					**<0.001**			0.248
Multifocal	27 (56.3%)	0 (0%)	15 (83.3%)	0 (0%)		0.496	0.201–1.227	
Vaginal pain	8 (16.7%)	3 (17.6%)	2 (11.1%)	6 (27.3%)		1.431	0.339–6.044	
Vulvar pain	6 (12.5%)	5 (29.4%)	1 (5.6%)	4 (18.2%)		-	-	
Vestibular pain	3 (6.3%)	6 (35.3%)	0 (0%)	4 (18.2%)		0.668	0.162–2.743	
Clitoral pain	0 (0%)	1 (5.9%)	0 (0%)	6 (27.3%)		0.517	0.077–3.452	
Perineal pain	3 (6.3%)	1 (5.9%)	0 (0%)	1 (4.5%)		1.460	0.354–6.028	
Urethral meatus	1 (2.1%)	0 (0%)	0 (0%)	1 (4.5%)		2.886	0.499–16.692	
Old obstetric scar	0 (0%)	1 (5.9%)	0 (0%)	0 (0%)		-	-	
CS	19 (39.6%)	6 (35.3%)	5 (27.8%)	5 (22.7%)	0.525	-	-	-
No CS	29 (60.4%)	11 (64.7%)	13 (72.2%)	17 (77.3%)	0.525	-	-	-
Comorbidities	17 (35.4%)	3 (17.6%)	2 (11.1%)	3 (13.6%)	0.079	1.492	0.729–3.005	0.274
Dyspareunia	30 (62.5%)	13 (76.5%)	17 (94.4%)	21 (95.5%)	**0.028**	0.740	0.354–1.547	0.423
Pain with micturition	18 (37.5%)	6 (35.2%)	6 (33.3%)	6 (27.3%)	0.844	-	-	-
Pain with defecation	15 (31.3%)	1 (5.9%)	2 (11.1%)	1 (4.5%)	**0.012**	1.438	0.622–3.323	0.396
Pain in pelvic trigger points	47 (97.9%)	17 (100%)	15 (83.3%)	21 (95.5%)	0.065	0.119	0.019–0.728	**0.006**

Abbreviations: CS: central sensitization; N: number of patients (%); HR: hazard ratio; CI 95%: confidence interval 95%; UA: univariate analysis; MA: multivariate analysis; *p*: statistical significance PP score: Convergences PP Score; GSV: generalized and spontaneous vulvodynia; LSV: localized and provoked vulvodynia; GPV: generalized and provoked vulvodynia; LPV: localized and spontaneous vulvodynia. Bold: significant number.

**Table 3:** Correlation between variables and treatment.

Demographics	Overall N = 105	CS N = 35	No CS N = 70	UA *p*	HR	CI 95%	MA *p*
Number of previous treatments, mean (range)	1.2 (0–7)	1.3	1.1	0.369	-	-	-
Number of prescribed treatments, mean (range)	2.8 (0–10)	3.4	2.4	**0.018**	0.847	0.714–1.004	0.056
Prescribed treatment, N	97 (92.4%)	35 (100%)	62 (88.6%)	**0.050**	3.297	0.984–11.046	0.053
Hygienic measures	10 (9.5%)	3 (8.6%)	7 (10.0%)		-	-	-
Gabapentin	3 (2.9%)	1 (2.9%)	2 (2.9%)	1.000	-	-	-
Pregabalin	11 (10.5%)	5 (14.3%)	6 (8.6%)	1.000	-	-	-
Duloxetine	11 (10.5%)	2 (5.7%)	9 (12.6%)	0.500	-	-	-
Amitriptyline	52 (49.5%)	21 (60.0%)	31 (44.3%)	0.329	-	-	-
Intravaginal diazepam	32 (30.5%)	12 (34.3%)	20 (28.6%)	0.129	-	-	-
Topical lidocaine	49 (46.7%)	28 (46.7%)	21 (60.0%)	0.549	-	-	-
Minimally invasive neuromodulation	33 (31.4%)	17 (48.5%)	16 (22.9%)	**0.007**	1.121	0.518–2.429	0.771
Psychological therapy	17 (16.2%)	10 (28.6%)	7 (10.0%)	**0.015**	0.863	0.308–2.423	0.780
Physiotherapy	75 (71.4%)	25 (71.4%)	50 (71.4%)	1.000	-	-	-
Physiotherapy, number sessions attended	3.9 (0–24)	4.3	3.8	0.657	-	-	-

Abbreviations: CS: central sensitization; N: number of patients (%); HR: hazard ratio; CI 95%: confidence interval 95%, UA: univariate analysis; MA: multivariate analysis; *p*: statistical significance. Bold: significant number.

**Table 4:** Treatment prescribed according to variables.

	Diazepam N = 32	Lidocaine N = 49	Amitryptiline N = 52	Anticonvulsant N = 14	MIN N = 33	Psych. N = 17	Physiotherapy N = 75
Type, *p*	**0.049**	0.077	**<0.001**	0.221	**0.021**	0.831	0.733
GSV	20 (62.5%)	17 (34.7%)	31 (59.6%)	8 (57.1%)	22 (66.7%)	7 (41.2%)	33 (44.0%)
LSV	1 (3.1%)	9 (18.4%)	13 (25.0%)	4 (28.6%)	5 (15.2%)	4 (23.5%)	14 (18.7%)
GPV	5 (15.6%)	8 (16.3%)	2 (3.8%)	1 (7.1%)	3 (9.1%)	3 (17.6%)	13 (17.3%)
LPV	6 (18.7%)	15 (30.6%)	6 (11.5%)	1 (7.1%)	3 (9.1%)	3 (17.6%)	15 (20.0%)
Pain location, *p*	0.237	**0.005**	0.093	0.917	0.417	0.080	0.502
Multifocal	16 (50.0%)	14 (28.6%)	19 (36.5%)	6 (42.9%)	17 (51.5%)	8 (47.1%)	30 (40%)
Vaginal pain	7 (21.9%)	7 (14.3%)	6 (11.5%)	1 (7.1%)	3 (9.1%)	2 (11.8%)	12 (16%)
Vulvar pain	3 (9.4%)	9 (18.4%)	9 (17.3%)	3 (17.6%)	5 (15.2%)	0 (0%)	12 (16%)
Vestibular pain	2 (6.3%)	12 (24.5%)	9 (17.3%)	2 (11.8%)	5 (15.2%)	2 (11.8%)	8 (10.7%)
Clitoral pain	1 (3.1%)	3 (6.1%)	5 (9.6%)	1 (7.1%)	1 (3.0%)	1 (5.9%)	5 (6.7%)
Perineal pain	3 (9.4%)	1 (2.0%)	1 (1.9%)	1 (7.1%)	2 (6.1%)	2 (11.8%)	5 (6.7%)
Urethral meat.	0 (0%)	2 (4.1%)	2 (3.8%)	0 (0%)	0 (0%)	1 (5.9%)	2 (2.7%)
Old obstet. scar	0 (0%)	1 (2.0%)	1 (3.8%)	0 (0%)	0 (0%)	1 (5.9%)	1 (1.3%)
Comorbidities, *p*	0.236	0.444	0.861	0.261	0.290	0.115	0.346
	10 (31.3%)	10 (20.4%)	12 (23.1%)	5 (35.7%)	10 (30.3%)	7 (41.2%)	16 (21.3%)
Dyspareunia, *p*	0.912	0.245	**0.012**	0.511	0.466	0.764	0.104
	24 (75.0%)	39 (79.6%)	33 (63.5%)	12 (85.7%)	23 (69.7%)	12 (70.6%)	59 (78.7%)
Pain with micturition, *p*	0.631	**<0.001**	0.303	0.193	0.390	0.082	0.659
	10 (31.3%)	25 (51.0%)	21 (40.4%)	7 (50.0%)	13 (39.4%)	9 (52.9%)	25 (33.3%)
Pain with defecation, *p*	**0.022**	**0.002**	0.446	0.069	**0.022**	0.508	0.691
	10 (31.3%)	3 (6.1%)	8 (15.4%)	6 (35.2%)	10 (30.3%)	4 (23.5%)	13 (17.3%)
Pain in pelvic trigger points, *p*	0.639	0.662	1.000	1.000	0.322	1.000	0.622
	30 (93.8%)	46 (93.9%)	50 (96.2%)	14 (100%)	33 (100%)	16 (94.1%)	72 (96%)
CS, *p*	0.549	**0.050**	0.129	0.543	**0.007**	**0.015**	1.000
	12 (37.5%)	21 (42.9%)	21 (40.3%)	6 (42.9%)	17 (51.5%)	10 (58.8%)	25 (33.3%)

Abbreviations: CS: central sensitization; N: number of patients (%); *p*: statistical significance PP score: Convergences PP Score; GSV: generalized and spontaneous vulvodynia; LSV: localized and provoked vulvodynia; GPV: generalized and provoked vulvodynia; LPV: localized and spontaneous vulvodynia; MIN: minimally invasive neuromodulation; Psych.: psychological therapy. Bold: significant number.

**Table 5:** Multivariate analysis of response to treatment.

	Response to Treatment (N = 51)			Days until Response	
	N (%)	Response (%)	*p*	Mean (Interval)	*p*
CS (N = 28)	16 (57.1%)	60.9%	0.073	293.4 (146.1–440.6)	0.073
No CS (N = 52)	35 (67.3%)	72.7%		228.8 (114.8–342.7)	
Pain with micturition (N = 31)	18 (58%)	64.4%	0.273	272.6 (127–418.2)	0.263
No pain with micturition (N = 49)	33 (67.3%)	71.5%		36 (124.3–347.6)	
Pain with defecation (N = 14)	5 (35.7%)	59.0%	0.284	180.8 (116.6–245)	0.447
No pain with defecation (N = 66)	46 (69.7%)	70.1%		244.7 (156.4–333)	
Dyspareunia (N = 59)	36 (61.0%)	66.7%	0.236	229.4 (139.9–318.4)	0.211
No dyspareunia (N = 21)	15 (71.4%)	74.7%		283.4 (88.9–477.8)	
Comorbidities (N = 59)	35 (59.3%)	66.4%	0.213	237.1 (143.6–330.6)	0.113
No comorbidities (N = 21)	16 (76.1%)	74.7%		246.8 (74.3–419.4)	
Vulvodynia Type			0.394		0.941
GSV (N = 37)	26 (70.2%)	66.5%		308.8 (205.2–412.4)	
LSV (N = 12)	7 (58.3%)	73.6%		327.7 (171.9–510.8)	
GPV (N = 14)	8 (57.1%)	63.1%		341.4 (163.7–378.3)	
LPV (N = 17)	9 (52.9%)	78.9%		271.0 (239.7–380.0)	

Abbreviations: CS: central sensitization; N: number of patients (%); *p*: statistical significance; GSV: generalized and spontaneous vulvodynia; LSV: localized and provoked vulvodynia; GPV: generalized and provoked vulvodynia; LPV: localized and spontaneous vulvodynia.

## Data Availability

Data presented in this manuscript are available from the corresponding authors on reasonable request.

## References

[1] Bornstein J., Goldstein A.T., Stockdale C.K., Bergeron S., Pukall C., Zolnoun D., Coady D. (2016). Consensus vulvar pain terminology committee of the International Society for the Study of Vulvovaginal Disease (ISSVD), the International Society for the Study of Women’s Sexual Health (ISSWSH), and the International Pelvic Pain Society (IPPS) 2015 ISSVD, ISSWSH and IPPS Consensus Terminology and Classification of Persistent Vulvar Pain and Vulvodynia. Obstet. Gynecol..

[2] Brotto L.A., Sadownik L.A., Thomson S., Dayan M., Smith K.B., Seal B.N., Moses M., Zhang A. (2014). A Comparison of Demographic and Psychosexual Characteristics of Women with Primary versus Secondary Provoked Vestibulodynia. Clin. J. Pain.

[3] Spadt S.K., Kingsberg S. Vulvar Pain of Unknown Cause (Vulvodynia): Clinical Manifestations and Diagnosis. UpToDate. https://www.uptodate.com/contents/vulvar-pain-of-unknown-cause-vulvodynia-clinical-manifestations-and-diagnosis.

[4] Gómez I., Coronado P.J., Martín C.M., Alonso R., Guisasola-Campa F.J. (2019). Study on the Prevalence and Factors Associated to Vulvodynia in Spain. Eur. J. Obstet. Gynecol. Reprod. Biol..

[5] Wojcik M., Plagens-Rotman K., Merks P., Mizgier M., Kedzia W., Jarzabek-Bielecka G. (2022). Visceral Therapy in Disorders of the Female Reproductive Organs. Ginekol. Pol..

[6] Sadownik L.A. (2000). Clinical Profile of Vulvodynia Patients. A prospective study of 300 patients. J. Reprod. Med..

[7] Levesque A., Riant T., Ploteau S., Rigaud J., Labat J.-J. (2018). Convergences PP Network Clinical Criteria of Central Sensitization in Chronic Pelvic and Perineal Pain (Convergences PP Criteria): Elaboration of a Clinical Evaluation Tool Based on Formal Expert Consensus. Pain Med..

[8] Woolf C.J. (1983). Evidence for a Central Component of Post-Injury Pain Hypersensitivity. Nature.

[9] Woolf C.J. (2007). Central Sensitization: Uncovering the Relation between Pain and Plasticity. Anesthesiology.

[10] Ji R.-R., Nackley A., Huh Y., Terrando N., Maixner W. (2018). Neuroinflammation and Central Sensitization in Chronic and Widespread Pain. Anesthesiology.

[11] Origoni M., Leone Roberti Maggiore U., Salvatore S., Candiani M. (2014). Neurobiological Mechanisms of Pelvic Pain. Biomed Res. Int..

[12] Torres-Cueco R., Nohales-Alfonso F. (2021). Vulvodynia—It Is Time to Accept a New Understanding from a Neurobiological Perspective. Int. J. Environ. Res. Public Health.

[13] Matzumura-Kasano J.P., Gutiérrez-Crespo H.F., Zamudio-Eslava L.A. (2018). Vulvodinia: Una Puesta Al Día. An. Fac. Med..

[14] Shah M., Hoffstetter S. (2014). Vulvodynia. Obstet. Gynecol. Clin. N. Am..

[15] Yunus M.B. (2008). Central Sensitivity Syndromes: A New Paradigm and Group Nosology for Fibromyalgia and Overlapping Conditions, and the Related Issue of Disease versus Illness. Semin. Arthritis Rheum..

[16] Pereira A., Fuentes L., Almoguera B., Chaves P., Vaquero G., Perez-Medina T. (2022). Understanding the Female Physical Examination in Patients with Chronic Pelvic and Perineal Pain. J. Clin. Med. Res..

[17] Spadt S.K., Kingsberg S. Vulvar Pain of Unknown Cause (Vulvodynia): Treatment. UpToDate. https://www.uptodate.com/contents/vulvar-pain-of-unknown-cause-vulvodynia-treatment.

[18] Wójcik M., Szczepaniak R., Placek K. (2022). Physiotherapy Management in Endometriosis. Int. J. Environ. Res. Public Health.

[19] Wojcik M., Jarzabek-Bielecka G., Merks P. (2022). The role of visceral therapy, Kegel’s muscle, core stability and diet in pelvic support disorders and urinary incontinence—Including sexological aspects and the role of physiotherapy and osteopathy. Ginekol. Pol..

[20] Whitney M., Papermaster A.E., Baum A., Wright M.L. (2022). Vulvodynia Is Not Associated with Concurrent Candidal Vaginitis. Women’s Health Rep..

[21] Hosmer D.W., Lemeshow L., Sturdivant R.X. (2013). Applied Logistic Regression.

[22] Reed B.D., Harlow S.D., Sen A., Legocki L.J., Edwards R.M., Arato N., Haefner H.K. (2012). Prevalence and Demographic Characteristics of Vulvodynia in a Population-Based Sample. Am. J. Obstet. Gynecol..

[23] Gumus I.I., Sarifakioglu E., Uslu H., Turhan N.O. (2008). Vulvodynia: Case Report and Review of Literature. Gynecol. Obstet. Investig..

[24] Pereira A., Pérez-Medina T., Rodríguez-Tapia A., Rutherford S., Millan I., Iglesias E., Ortiz-Quintana L. (2014). Chronic Perineal Pain: Analyses of Prognostic Factors in Pudendal Neuralgia. Clin. J. Pain.

[25] Patla G., Mazur-Bialy A.I., Humaj-Grysztar M., Bonior J. (2023). Chronic Vulvar Pain and Health-Related Quality of Life in Women with Vulvodynia. Life.

[26] Pereira G.M.V., Marcolino M.S., Reis Z.S.N., Monteiro M.V.D.C. (2018). A systematic review of drug treatment of vulvodynia: Evidence of a strong placebo effect. BJOG Int. J. Obstet. Gynaecol..

